# Skin Microbiota: Mediator of Interactions Between Metabolic Disorders and Cutaneous Health and Disease

**DOI:** 10.3390/microorganisms13010161

**Published:** 2025-01-14

**Authors:** Magdalini Kreouzi, Nikolaos Theodorakis, Maria Nikolaou, Georgios Feretzakis, Athanasios Anastasiou, Konstantinos Kalodanis, Aikaterini Sakagianni

**Affiliations:** 1 Department of Internal Medicine, Amalia Fleming General Hospital, 14, 25th Martiou Str., 15127 Athens, Greece; kreouzi.m@live.unic.ac.cy; 2 NT-CardioMetabolics, Clinic for Metabolism and Athletic Performance, 47 Tirteou Str., 17564 Palaio Faliro, Greece; n.theodorakis@flemig-hospital.gr; 3 Department of Cardiology & Preventive Cardiology Outpatient Clinic, Amalia Fleming General Hospital, 14, 25th Martiou Str., 15127 Melissia, Greece; m.nikolaou@flemig-hospital.gr; 4 School of Medicine, National and Kapodistrian University of Athens, 75 Mikras Asias, 11527 Athens, Greece; 5 School of Science and Technology, Hellenic Open University, 18 Aristotelous Str., 26335 Patras, Greece; 6 Biomedical Engineering Laboratory, National Technical University of Athens, 15780 Athens, Greece; aanastasiou@biomed.ntua.gr; 7 Department of Informatics & Telematics, Harokopio University of Athens, 17676 Kallithea, Greece; kkalodanis@hua.gr; 8 Intensive Care Unit, Sismanogleio General Hospital, 37 Sismanogleiou Str., 15126 Marousi, Greece

**Keywords:** metabolic disorders, skin microbiota, biomarkers, atopic dermatitis, psoriasis, acne, precision dermatology

## Abstract

Metabolic disorders, including type 2 diabetes mellitus (T2DM), obesity, and metabolic syndrome, are systemic conditions that profoundly impact the skin microbiota, a dynamic community of bacteria, fungi, viruses, and mites essential for cutaneous health. Dysbiosis caused by metabolic dysfunction contributes to skin barrier disruption, immune dysregulation, and increased susceptibility to inflammatory skin diseases, including psoriasis, atopic dermatitis, and acne. For instance, hyperglycemia in T2DM leads to the formation of advanced glycation end products (AGEs), which bind to the receptor for AGEs (RAGE) on keratinocytes and immune cells, promoting oxidative stress and inflammation while facilitating Staphylococcus aureus colonization in atopic dermatitis. Similarly, obesity-induced dysregulation of sebaceous lipid composition increases saturated fatty acids, favoring pathogenic strains of *Cutibacterium acnes*, which produce inflammatory metabolites that exacerbate acne. Advances in metabolomics and microbiome sequencing have unveiled critical biomarkers, such as short-chain fatty acids and microbial signatures, predictive of therapeutic outcomes. For example, elevated butyrate levels in psoriasis have been associated with reduced Th17-mediated inflammation, while the presence of specific Lactobacillus strains has shown potential to modulate immune tolerance in atopic dermatitis. Furthermore, machine learning models are increasingly used to integrate multi-omics data, enabling personalized interventions. Emerging therapies, such as probiotics and postbiotics, aim to restore microbial diversity, while phage therapy selectively targets pathogenic bacteria like *Staphylococcus aureus* without disrupting beneficial flora. Clinical trials have demonstrated significant reductions in inflammatory lesions and improved quality-of-life metrics in patients receiving these microbiota-targeted treatments. This review synthesizes current evidence on the bidirectional interplay between metabolic disorders and skin microbiota, highlighting therapeutic implications and future directions. By addressing systemic metabolic dysfunction and microbiota-mediated pathways, precision strategies are paving the way for improved patient outcomes in dermatologic care.

## 1. Introduction

Metabolic disorders, including type 2 diabetes mellitus (T2DM), obesity, and metabolic syndrome (MetS), pose significant global health challenges due to their systemic effects and numerous comorbidities [1]. These conditions, characterized by disrupted glucose and lipid metabolism, insulin resistance, and chronic low-grade inflammation (meta-inflammation), have traditionally been linked to cardiovascular–renal–hepatic diseases [2]. However, emerging evidence highlights their far-reaching impact on the skin, particularly through interactions with the skin microbiota, a dynamic ecosystem critical for cutaneous health [3].

The skin microbiota, comprising bacteria (e.g., *Staphylococcus epidermidis*, *Cutibacterium acnes*), fungi (e.g., *Malassezia* spp.), viruses, and mites, is essential for cutaneous health [4]. This microbial community plays a pivotal role in modulating immune responses, maintaining epidermal barrier integrity, and competing with pathogenic organisms. Metabolic dysregulation alters these interactions at molecular and cellular levels. For instance, hyperglycemia-driven advanced glycation end products (AGEs) compromise skin barrier proteins, while imbalances in adipokines and cytokines in obesity shift immune responses and modify epidermal lipid composition, reshaping microbial communities [5,6]. These changes exacerbate susceptibility to dermatologic conditions such as psoriasis, atopic dermatitis (AD), and chronic wounds [7,8,9].

Additionally, with the rise of integrative ‘omics’ and large-scale clinical data, machine learning (ML) approaches are increasingly being employed to identify complex patterns, predict disease risk, and evaluate therapeutic responses. Such computational tools can integrate data streams from metabolic profiling, microbiome sequencing, and clinical outcomes, helping to unravel the intricate interplay between metabolic disorders, the skin microbiome, and dermatologic health [10,11].

This review explores the bidirectional interactions between metabolic dysfunction and the skin microbiota, focusing on their roles in barrier disruption, immune dysregulation, and dermatologic conditions such as psoriasis, AD, and acne. We highlight insights from omics technologies, including metabolomics and microbiomics, which have identified novel biomarkers predictive of disease risk and therapeutic outcomes. Emerging therapies, such as probiotics, postbiotics, and phage therapy, are examined for their potential to correct microbial dysbiosis while addressing underlying metabolic abnormalities. Advances in ML further enhance our ability to integrate complex multi-omics data, driving innovations in precision dermatology.

Regarding the topic of this manuscript, there is a significant gap in the literature, with only a few studies briefly referencing interactions between microbiomics and metabolic dysregulation within the broader pathophysiology of certain skin diseases. This review uniquely focuses on the interplay between metabolic disorders, the skin microbiota, and cutaneous health and disease. By addressing this critical gap, this manuscript provides a fresh perspective on integrated strategies to improve dermatologic outcomes in patients with metabolic disorders.

## 2. Methods

This manuscript is a narrative literature review synthesizing current knowledge on the role of the skin microbiota as a mediator of interactions between metabolic disorders and skin health. A comprehensive literature search was conducted in PubMed and Scopus for studies published up to 22 December 2024. We utilized a wide range of keywords and Medical Subject Headings (MeSH) terms to ensure a thorough search, including but not limited to skin microbiota, metabolic disorders, obesity, diabetes mellitus, dysbiosis, skin diseases, psoriasis, atopic dermatitis, microbial interactions, and ML. Boolean operators were applied to refine and optimize the search strategy.

The inclusion criteria were as follows:

▪ Study Type: Reviews or original research articles published in English.
▪ Focus: Articles that addressed metabolic and immunological factors influencing the skin microbiota and its role in skin diseases.
▪ Therapeutic Studies: Relevant therapeutic studies were defined as those investigating microbiota-targeted interventions (e.g., probiotics, postbiotics, or phage therapy) and/or systemic treatments addressing underlying metabolic dysfunction. These studies were assessed for clinical outcomes, mechanistic insights, and validation through preclinical or clinical data.
▪ ML Applications: Key records describing the application of ML tools or algorithms in integrating omics datasets (e.g., metabolomics, microbiomics) and predicting disease outcomes or therapeutic responses were included. Specific ML approaches, such as supervised learning algorithms (e.g., random forests, support vector machines) or unsupervised clustering methods, were highlighted for their contributions to advancing precision dermatology.

The literature search was performed independently by two authors to ensure objectivity and thoroughness. Any inconsistencies in study selection or data extraction were resolved by consensus with a third author. Articles were initially screened based on their titles and abstracts to assess relevance, followed by full-text reviews for those meeting the inclusion criteria. Additional relevant studies were identified through manual reviews of reference lists from selected articles. The findings were synthesized to present an integrated and up-to-date perspective on the bidirectional interactions between metabolic disorders, skin microbiota, and dermatologic health, along with therapeutic and computational advancements in the field.

## 3. Metabolic Disorders Overview

Metabolic disorders, including T2DM, obesity, and MetS, are characterized by intricate interactions among genetic predispositions, environmental exposures, and lifestyle habits. These conditions disrupt normal metabolic and immune processes, resulting in systemic effects that extend to various tissues, including the skin. There is significant overlap among specific skin diseases and metabolic disorders:

▪ The prevalence of MetS is estimated to range between 20% and 50% among individuals with psoriasis, with higher rates observed in those with more severe forms of the disease [12].
▪ The prevalence of MetS in patients with lichen planus (LP) varies in published studies, ranging from 6% to 77% [13].
▪ A cross-sectional study involving 116,816 patients with AD and 116,812 control participants revealed that moderate and severe AD were linked to significantly higher prevalence rates of MetS (17.0% vs. 9.4%) and its individual components, including obesity (22.2% vs. 18.6%), diabetes (15.9% vs. 9.2%), hypertension (27.9% vs. 15.3%), and dyslipidemia (47.1% vs. 28.5%) (all *p* < 0.001) [14].
▪ The literature indicates that patients with hidradenitis suppurativa (HS) have an estimated odds ratio (OR) of 2.66 (95% CI: 1.90–3.72) for developing MetS [15].
▪ A small study found that MetS was present in 35.2% of patients with seborrheic dermatitis (SD) compared to 10.6% of the control group [16]. However, another small study observed that only high-density lipoprotein cholesterol (HDL-C) levels were significantly lower in the patient group compared to the control group [17].
▪ According to a recent meta-analysis, MetS has been strongly associated with specific skin diseases, including HS (OR: 4.46), LP (OR: 3.79), and SD (OR: 2.45). Psoriasis also showed a significant correlation with MetS, although with high heterogeneity (OR: 2.89). In contrast, rosacea exhibited a weaker association with MetS, with an odds ratio (OR) of 1.56 (95% CI: 0.96–2.52). Nonetheless, a significant relationship was observed between rosacea and high blood pressure (OR: 1.204, 95% CI: 1.09–1.33) as well as insulin resistance (OR: 2.33, 95% CI: 1.18–4.60) [18].

A closer look at their molecular mechanisms reveals how these disorders drive chronic inflammation and metabolic dysfunction.

### 3.1. T2DM

▪ Core Mechanisms of T2DM [19,20]:
  o Progressive insulin resistance and declining insulin secretion are hallmarks.
  o Insulin resistance originates from defects in insulin receptor signaling, particularly at the level of insulin receptor substrate-1 (IRS-1).
  o Impaired IRS-1 phosphorylation disrupts interaction with phosphoinositide 3-kinase (PI3K), a critical enzyme for glucose uptake.
  o Dysfunctional PI3K prevents activation of Akt (protein kinase B), inhibiting GLUT4 translocation to the cell membrane and reducing glucose uptake in skeletal muscle and adipocytes.
▪ Role of Hyperglycemia and Glucotoxicity [19,20]:
  o Chronic hyperglycemia exacerbates insulin resistance through glucotoxicity.
  o High glucose levels increase the mitochondrial production of reactive oxygen species (ROS), which damage IRS proteins and β-cells, further impairing insulin sensitivity and secretion.
▪ Systemic Metabolic Inflammation in T2DM (meta-inflammation) [19,20]:
  o Mediated by pro-inflammatory cytokines largely produced by dysfunctional adipose tissue, such as tumor necrosis factor-alpha (TNF-α) and interleukin-6 (IL-6).
  o TNF-α induces serine phosphorylation of IRS-1, impairing insulin receptor signaling.
  o IL-6 disrupts insulin sensitivity via the janus kinase/signal transducers and activators of transcription (JAK/STAT) pathways.
▪ Advanced Glycation End Products (AGEs) and RAGE Pathway [19,20,21,22]:
  o Prolonged hyperglycemia in metabolic disorders leads to the non-enzymatic glycation of proteins and lipids, forming AGEs.
  o These molecules engage RAGE, a receptor expressed on immune and endothelial cells, triggering several downstream effects.
  o AGE-RAGE interactions stimulate the production of ROS through nicotinamide adenine dinucleotide phosphate (NADPH) oxidase, amplifying oxidative stress.
  o The resulting activation of NF-κB promotes the transcription of pro-inflammatory cytokines, such as TNF-α and IL-1β, and vascular adhesion molecules, facilitating leukocyte recruitment and chronic inflammation.
  o In the skin, AGEs have profound effects on structure and function. They cross-link collagen and elastin fibers in the extracellular matrix, reducing skin elasticity and barrier integrity.
  o AGEs also impair keratinocyte differentiation and antimicrobial peptide (AMP) production, leaving the skin more susceptible to infections.
  o This cascade is particularly pronounced in individuals with T2DM, where AGE accumulation is linked to complications such as poor wound healing, chronic ulcers, and heightened susceptibility to microbial infections.

### 3.2. Obesity

▪ Core Characteristics [23,24,25]:
  o Obesity is characterized by excessive and dysfunctional adipose tissue accumulation, which acts as an active endocrine organ.
  o Adipose tissue secretes hormones, cytokines, and adipokines that regulate metabolism and immune responses.
▪ Inflammatory Response [23,24,25]:
  o Adipose tissue expansion leads to hypoxia, triggering macrophage infiltration.
  o Infiltrating macrophages adopt a pro-inflammatory M1 phenotype, releasing cytokines such as TNF-α, IL-1β, and IL-6.
▪ Adipokine Dysregulation [23,24,25]:
  o Dysregulated secretion of adipokines, including elevated leptin and resistin, contributes to inflammation and metabolic dysfunction.
  o Leptin: Promotes inflammation via activation of the JAK/STAT signaling pathway.
  o Resistin: Exacerbates insulin resistance by interacting with Toll-like receptor-4 (TLR-4).
▪ Lipid Metabolism Dysregulation [23,24,25]:
  o Excess circulating free fatty acids activate protein kinase C (PKC), impairing insulin receptor signaling and exacerbating systemic insulin resistance.
  o Lipotoxicity damages pancreatic β-cells and hepatocytes, further compounding metabolic dysfunction.
▪ Chronic Low-Grade Inflammation (Meta-Inflammation) [23,24,25]:
  o A systemic inflammatory state driven by dysregulated adipokines and cytokines.
  o This inflammatory milieu affects multiple organs and promotes insulin resistance and obesity-related complications.

### 3.3. Metabolic Syndrome

▪ Overview [26,27]:
  o MetS comprises central obesity, hypertension, dyslipidemia, and insulin resistance.
  o These interrelated conditions collectively increase the risk of cardiovascular diseases, T2DM, and systemic complications.
▪ Visceral Adiposity [26,27]:
  o Central to MetS is visceral adiposity, with fat accumulation around abdominal organs.
  o This fat depot acts as an endocrine organ, secreting the following:
    - Elevated levels of pro-inflammatory adipokines, such as leptin, resistin, and visfatin.
    - Reduced levels of anti-inflammatory adiponectin.
  o This imbalance induces chronic low-grade inflammation (meta-inflammation), driving systemic immune activation and exacerbating metabolic dysfunction.
▪ Pro-Inflammatory Signaling Pathways [26,27]:
  o Pro-inflammatory adipokines and cytokines, including TNF-α and IL-6, activate key signaling pathways: NF-κB and JAK/STAT.
  o These pathways disrupt insulin signaling, alter macrophage polarization, and impair endothelial function, creating a feedback loop that perpetuates metabolic and cardiovascular risks.
▪ Dyslipidemia [26,27]:
  o Features include elevated triglycerides, HDL, and small dense low-density lipoprotein (LDL) particles.
  o Consequences of lipid imbalance:
    - Oxidized LDL infiltrates vascular walls, triggering macrophage recruitment and foam cell formation, key events in atherogenesis.
    - Reduced HDL-C impairs reverse cholesterol transport, limiting the removal of cholesterol from atherosclerotic plaques.
▪ Hypertension and Vascular Dysfunction [26,27]:
  o Hypertension exacerbates risks by reducing nitric oxide (NO) bioavailability, driven by oxidative stress and impaired endothelial NO synthase activity.
  o Outcomes include vasoconstriction, endothelial activation, vascular remodeling, arterial stiffness, and hypertension.
▪ Combined Impact: These interconnected conditions create a highly atherogenic and pro-inflammatory environment, predisposing individuals to complications such as coronary artery disease, stroke, heart failure with preserved ejection fraction, chronic kidney disease, and metabolic dysfunction-associated steatotic liver disease [25,26].

## 4. Skin Microbiota: A Key Mediator of Cutaneous Health

The skin microbiota represents a complex and dynamic community of microorganisms, including bacteria, fungi, viruses, and mites, which inhabit the diverse ecological niches of the skin. These microorganisms are not randomly distributed but are shaped by variations in the skin’s biophysical properties, such as pH, moisture levels, lipid content, and exposure to environmental factors. Sebaceous areas, for example, are rich in lipids and promote the growth of lipophilic organisms like *C. acnes*, which metabolizes sebum triglycerides into free fatty acids. Moist areas, such as the axillae, host *Corynebacterium* species, while dry regions predominantly harbor *S. epidermidis* and other resilient bacteria. Fungi, such as species from the genus *Malassezia*, are also common in sebaceous areas, while other microbial communities vary across different body sites and skin types [4].

The skin microbiota plays an essential role in maintaining skin homeostasis and preventing disease. Commensal microorganisms interact with the host to perform critical functions, such as fortifying the skin barrier, modulating immune responses, and outcompeting potential pathogens. For instance, *S. epidermidis* produces AMPs, such as phenol-soluble modulins, which inhibit the colonization of pathogenic bacteria like *Staphylococcus aureus*. Similarly, *C. acnes* can regulate the skin’s pH by hydrolyzing sebum triglycerides, contributing to an acidic environment unfavorable to pathogens. Beyond these chemical defenses, commensals also influence physical barrier integrity by promoting keratinocyte differentiation and lipid production, essential for the formation of the stratum corneum [28].

The interaction between the skin microbiota and the host immune system is another critical component of skin health. Commensals engage with host pattern recognition receptors (PRRs), such as TLRs and nucleotide-binding oligomerization domain (NOD)-like receptors, to modulate immune responses. These interactions ensure a balance between immune tolerance to commensals and activation against invading pathogens. For example, *S. epidermidis* can activate TLR2 on keratinocytes to induce AMP production while also promoting regulatory T-cell (Treg) activity, which prevents excessive inflammation. These immune-regulating functions of the microbiota are crucial for maintaining a balanced immune tone in the skin, reducing the likelihood of inflammatory and autoimmune conditions [29].

Disruptions in the skin microbiota, known as dysbiosis, are implicated in a range of skin diseases. Dysbiosis can arise from factors such as antibiotic use, harsh skincare products, genetic predispositions, or underlying immune dysfunction. In inflammatory conditions like AD, dysbiosis is mostly characterized by an overgrowth of *S. aureus*, which exacerbates inflammation and disrupts the epidermal barrier. Psoriasis is another condition linked to dysbiosis, characterized by additional shifts in the microbial composition that may amplify T-helper (Th)17-mediated inflammatory responses. Specifically, in psoriasis, there is an increase in *S. aureus* and *S. pyogenes* in plaques. Furthermore, psoriasis shows a reduced presence of commensal bacteria such as *Cutibacterium* and *Corynebacterium*. Similarly, acne vulgaris is associated with changes in the abundance and activity of *C. acnes*, where certain strains may overproduce pro-inflammatory lipids and contribute to follicular inflammation [30].

Fungal and viral components of the microbiota also play significant roles in skin health and disease. *Malassezia* species, while commensal in healthy skin, are associated with conditions such as SD and pityriasis versicolor when their growth becomes uncontrolled. Viruses, such as human papillomaviruses and herpesviruses, can remain dormant in the skin but may become pathogenic under conditions of immunosuppression or barrier disruption. The interplay between microbial communities, the host immune system, and external factors defines the balance between health and disease [30].

The composition of the skin microbiota undergoes significant changes with aging, influenced by shifts in host factors such as sebaceous gland activity, skin lipids, natural moisturizing factors (NMFs), and antimicrobial peptides (AMPs). A study examining 158 Caucasian females aged 20 to 74 found that bacterial diversity increases with age across the forearm, buttock, and facial skin. Notably, the abundance of Cutibacterium and Lactobacillus decreased with age at all sites, correlating with a reduction in sebaceous gland size and lipid production, which are critical for maintaining these bacteria. Conversely, genera such as Streptococcus and Anaerococcus showed site-specific increases, likely driven by shifts in the availability of skin lipids and other host factors. Increased NMFs and AMPs with age were positively correlated with bacterial genera like Corynebacterium and Finegoldia, highlighting how host changes influence microbial dynamics. These findings suggest that age-related alterations in skin biology drive the restructuring of microbial communities [31].

Race and ethnicity also significantly influence microbiota composition, with differences emerging as early as three months of age and persisting through childhood and adulthood. Variability in microbial taxa is linked to inequitable environmental and social factors, rather than biological differences. For example, specific taxa like Bifidobacterium and Lactobacillus are enriched in racial groups with higher breastfeeding rates, while other differences correlate with factors such as diet and hygiene. ML models using gut microbiome data from children have demonstrated an 87% accuracy in predicting caregiver-identified race and ethnicity, underscoring the role of structural disparities in shaping microbial communities. These findings emphasize the need for personalized approaches in microbiome research to address demographic variability and health disparities [32].

An overview of the commensal and pathogenic interactions of bacteria colonizing the skin and/or being part of the human skin flora is presented in Table 1.

## 5. Interactions Between Metabolic Disorders and the Skin Microbiota

Metabolic disorders, including T2DM and obesity, create a systemic environment of chronic low-grade inflammation, also known as meta-inflammation. This state profoundly affects the skin microbiota by altering the biochemical, structural, and immune landscape of the epidermis. Elevated levels of pro-inflammatory cytokines, such IL-6, TNF-α, and IL-1β, play pivotal roles in driving these changes. These cytokines, produced by hypertrophic adipocytes, immune cells, and the liver, directly influence keratinocyte gene expression by activating intracellular signaling pathways, including the NF-κB- and mitogen-activated protein kinase (MAPK) cascades [33,34].

### 5.1. Impact on Keratinocytes and the Epidermal Barrier

Keratinocytes, the primary cell type in the epidermis, rely on tightly regulated processes of proliferation, differentiation, and lipid synthesis to maintain the integrity of the stratum corneum. In metabolic disorders, elevated cytokine levels disrupt these processes by altering the expression of genes encoding structural proteins such as involucrin, loricrin, and filaggrin. For example, TNF-α and IL-1β activate the NF-κB pathway, leading to the suppression of involucrin and loricrin transcription. These proteins are essential for forming the cornified envelope, a critical component of the skin’s physical barrier. The reduction in filaggrin levels compromises the production of natural moisturizing factors, which are derived from filaggrin degradation and include hygroscopic molecules such as urocanic acid and pyrrolidone carboxylic acid. This depletion of natural moisturizing factors increases transepidermal water loss and desiccates the epidermis, setting the stage for microbial invasion [35,36].

Furthermore, IL-6 signaling through its receptor complex (IL-6R and gp130) activates the janus kinase/signal transducers and activators of transcription (JAK/STAT) pathway in keratinocytes, leading to the expression of pro-inflammatory genes and a shift in AMP production. Cathelicidins (e.g., LL-37) and β-defensins, crucial AMPs that maintain microbial balance, are downregulated, weakening the skin’s chemical defense against pathogens. The cumulative effect of these changes is a weakened epidermal barrier that is highly permeable to environmental insults and opportunistic microbes [35,36].

Emerging evidence highlights a critical link between glucose metabolism reprogramming in keratinocytes and the pathogenesis of psoriasis, particularly in the context of MetS. A recent study by Yan et al. (2024) demonstrated that psoriatic keratinocytes exhibit enhanced glycolysis, evidenced by elevated levels of glycolysis-related metabolites such as glucose-6-phosphate, fructose-1,6-bisphosphate, and lactate. In patients with psoriasis and MetS, glycolysis-related protein expression (e.g., GLUT1, HK2, and PFKFB3) was significantly upregulated, correlating with disease severity. Importantly, high-glucose and high-fat culture intensified this metabolic reprogramming through the AKT/mTOR pathway, exacerbating keratinocyte proliferation and inflammatory cytokine production. Glycolysis inhibition via agents like 2-deoxyglucose effectively attenuated these pathological features, offering potential therapeutic avenues [37].

Cathelicidin, an AMP, plays a dual role in innate immunity and systemic metabolic regulation. Studies have linked LL-37 to inflammation and insulin resistance in MetS. Activation of Toll-like receptors (e.g., TLR4) by LL-37 promotes pro-inflammatory cytokine secretion, disrupting adipocyte insulin signaling and lipid metabolism. Notably, LL-37 levels inversely correlate with HDL cholesterol while positively associating with triglycerides, highlighting its potential role in atherogenic dyslipidemia. Beyond metabolic regulation, LL-37 is implicated in psoriasis-related inflammation by modulating keratinocyte immune responses and promoting cytokine production. These findings underscore LL-37’s role as both a mediator of systemic inflammation and a biomarker for metabolic and inflammatory conditions [38].

### 5.2. RAGE Pathway

AGEs accumulate in individuals with diabetes and obesity due to prolonged hyperglycemia and oxidative stress. These molecules irreversibly cross-link proteins such as collagen and elastin, stiffening the extracellular matrix and impairing the biomechanical properties of the dermis. AGEs bind to RAGE, a PRR expressed on keratinocytes, endothelial cells, and immune cells. Activation of RAGE triggers the recruitment of intracellular adapter molecules such as myeloid differentiation primary response 88 (MyD88) and Toll–interleukin 1 receptor domain-containing adapter protein (TIRAP), initiating downstream signaling cascades. These cascades involve NF-κB and extracellular signal-regulated kinases (ERK1/2), resulting in the transcription of pro-inflammatory cytokines and matrix metalloproteinases (MMPs). MMP-9 and MMP-2 degrade the extracellular matrix, further disrupting the dermal architecture and facilitating microbial infiltration [21,22].

The interaction of AGEs with RAGE also generates ROS via the activation of NADPH oxidase, leading to oxidative damage of keratinocyte membranes and intracellular organelles. This oxidative stress reduces mitochondrial function and impairs ATP production, critical for keratinocyte proliferation and differentiation. Additionally, ROS interact with lipid peroxidation pathways, generating malondialdehyde and 4-hydroxynonenal, which further exacerbate cellular damage and impair lipid synthesis. These lipid alterations directly affect sebum composition and the skin’s ability to sustain commensal microbiota [21,22].

### 5.3. Alterations in Sebum Composition and Microbial Metabolism

Sebaceous glands, under the regulation of insulin and androgens, secrete a complex mixture of lipids, including triglycerides, wax esters, cholesterol, and squalene. In metabolic disorders, insulin resistance alters sebaceous gland function, skewing the lipid profile toward increased production of saturated fatty acids and decreased levels of unsaturated fatty acids. These lipid changes directly influence the metabolic activity of the microbiota. For instance, *C. acnes*, a dominant commensal in sebaceous-rich areas, relies on triglycerides and unsaturated fatty acids for its metabolic processes. A reduction in these substrates hampers the growth of commensal strains, while favoring opportunistic pathogens capable of metabolizing saturated fatty acids. Pathogenic strains of *C. acnes* produce porphyrins and short-chain fatty acids (SCFAs), which trigger the expression of inflammatory cytokines in keratinocytes via activation of aryl hydrocarbon receptors. This inflammatory feedback loop exacerbates skin conditions such as acne and folliculitis. Lipase and protease activity from resident and pathogenic microbes also modulate the stratum corneum’s permeability. Lipases hydrolyze sebum triglycerides into free fatty acids, which can disrupt tight junctions between keratinocytes. Proteases degrade corneodesmosomal proteins such as desmoglein-1 and corneodesmosin, weakening cell–cell adhesion and facilitating microbial penetration into deeper layers of the epidermis [39,40].

### 5.4. Immune Dysregulation and Microbial Dynamics

Chronic systemic inflammation associated with metabolic disorders disrupts immune surveillance in the skin. The trafficking and function of Langerhans cells and dermal dendritic cells are altered by the inflammatory milieu. TNF-α and IL-6 suppress the migration of Langerhans cells to draining lymph nodes, reducing their ability to present antigens to naïve T cells. Instead, these cells remain in the epidermis, where they promote the activation of effector T cells, particularly Th17 cells. The resultant production of IL-17 and IL-22 drives keratinocyte hyperproliferation and disrupts epidermal differentiation, creating conditions that favor dysbiosis [41].

These immune shifts also impair the skin’s ability to tolerate commensal microorganisms. For example, *S. epidermidis*, a commensal that normally promotes immune tolerance through the production of lipoteichoic acid, becomes less effective in modulating immune responses. The reduced Treg activity and increased Th1/Th17 polarization skew the immune environment toward an inflammatory state, further destabilizing microbial communities [41].

In inflammatory skin diseases, cytokines such as TNF-α and IL-6 play a dual role in influencing skin microbiota dynamics and being modulated by microbial shifts. For instance, in psoriasis, TNF-α and IL-6 are significantly upregulated, leading to enhanced keratinocyte proliferation and immune cell infiltration. These cytokines create an inflammatory milieu that disrupts microbial homeostasis, favoring pathogenic taxa like Staphylococcus aureus and Streptococcus pyogenes. Conversely, microbial dysbiosis exacerbates cytokine production. S. aureus releases superantigens like SEB, which activate dendritic cells to produce IL-12 and IL-23, further amplifying Th17-mediated cytokine cascades, including IL-17 and IL-22. These interactions exemplify a self-reinforcing loop where cytokine-driven inflammation alters microbial communities, and microbial products sustain and intensify cytokine responses, perpetuating chronic skin inflammation. Such findings highlight the importance of targeting both microbiota and cytokine pathways for therapeutic interventions [42].

### 5.5. Microvascular Impairment and Hypoxia

Microvascular complications, a hallmark of diabetes, significantly impact skin homeostasis. Hyperglycemia-induced endothelial dysfunction reduces nitric oxide (NO) bioavailability, impairing vasodilation and capillary perfusion. The resultant hypoxic conditions activate hypoxia-inducible factor 1-alpha (HIF-1α) in keratinocytes and sebocytes, altering their metabolic activity. HIF-1α upregulates glycolytic enzymes, shifting cellular metabolism toward anaerobic glycolysis. This metabolic shift reduces ATP availability and affects keratinocyte turnover, creating niches that favor anaerobic or facultative anaerobic microbes such as *C. acnes* and *Pseudomonas aeruginosa*. Reduced perfusion also delays the clearance of metabolic byproducts and toxins, further modifying the microbial habitat [43].

Sweat gland dysfunction, commonly observed in diabetic neuropathy, exacerbates these issues. Reduced sweat secretion alters ionic gradients and skin hydration, favoring the colonization of halophilic and xerophilic species such as *S. aureus* and certain fungi. Neural inputs that regulate microvascular tone and glandular activity are also disrupted, creating patchy areas of skin with distinct microbial communities. Over time, these microenvironmental disparities increase the risk of localized infections and biofilm formation [43].

## 6. Psoriasis

Psoriasis is a chronic immune-mediated skin condition characterized by erythematous, scaly plaques resulting from excessive keratinocyte proliferation and incomplete differentiation. At the core of its pathogenesis lies the IL-23/IL-17 axis, driven by Th17 cells and their cytokines, which create a pro-inflammatory environment. Recent evidence highlights how metabolic disorders, such as obesity and MetS, and disruptions in the skin microbiome interact to amplify the inflammatory pathways underlying psoriasis, forming a vicious cycle of immune activation and microbial dysbiosis [44].

Dendritic cells play a critical role in psoriasis by responding to environmental triggers, microbial antigens, and damage-associated molecular patterns like DNA and LL-37 complexes. These cells release IL-23, which is essential for maintaining Th17 cells. Th17 cells secrete IL-17A, IL-17F, and IL-22, which promote keratinocyte hyperproliferation and inhibit proper differentiation. These cytokines synergistically upregulate AMPs such as β-defensins and S100 proteins in keratinocytes, attracting neutrophils and intensifying inflammation. This process becomes exacerbated in the presence of MetS, as hypertrophic adipocytes and infiltrating macrophages secrete high levels of pro-inflammatory cytokines, including TNF-α, IL-6, and IL-1β. These cytokines activate NF-κB signaling in keratinocytes and immune cells, heightening inflammatory responses. Elevated leptin in obesity promotes Th17 cell polarization, while decreased adiponectin removes an anti-inflammatory control, further fueling psoriatic inflammation [45].

The microbiome in psoriasis is characterized by dysbiosis, marked by reduced microbial diversity and a shift toward pathogenic species. In psoriatic lesions, *S. aureus* and *Streptococcus pyogenes* are overrepresented and can act as superantigens, stimulating T-cell activation. Conversely, commensal bacteria like *Cutibacterium* and *Corynebacterium* are diminished, leading to weakened immune regulation. Dysbiosis amplifies inflammation through PRRs on keratinocytes, including TLRs (TLR2, TLR4) and NOD-like receptors, creating a feedback loop that sustains inflammation and further disrupts the microbiome [46].

Psoriatic plaques also exhibit altered lipid composition, including reduced ceramides, elevated free fatty acids, and increased cholesterol sulfate. These changes compromise the stratum corneum’s barrier integrity, increasing transepidermal water loss and exposing underlying layers to microbial invasion. *S. aureus* exacerbates this disruption by producing enterotoxins that activate IL-23 and IL-17 pathways, intensifying keratinocyte activation and neutrophilic infiltration.

Recent studies emphasize the role of lipid metabolism dysregulation in psoriasis and its potential therapeutic implications. For instance, a meta-analysis demonstrated significantly higher levels of total cholesterol, LDL, and triglycerides, with a marked reduction in HDL in psoriasis patients compared to controls (*p* < 0.05). These changes compromise the stratum corneum’s barrier integrity, increasing transepidermal water loss and exposing underlying layers to microbial invasion. Aberrant lipid profiles were further associated with elevated serum IL-6, which positively correlated with the LDL/HDL ratio (r = 0.48, *p* < 0.01). Dysregulated lipid metabolism contributes to microbial dysbiosis, where lipid-rich psoriatic plaques foster pathogenic taxa such as *Staphylococcus aureus* while depleting beneficial commensals of *Cutibacterium*. *S. aureus* exacerbates the epidermal barrier disruption by producing enterotoxins that activate IL-23 and IL-17 pathways, intensifying keratinocyte activation and neutrophilic infiltration. Fungal dysbiosis, particularly with *Malassezia* species, further contributes to psoriasis by degrading sebaceous lipids into free fatty acids, which irritate keratinocytes and drive dendritic cell and Th17 activation, especially in sebaceous-rich areas like the scalp [47,48].

Metabolic disorders amplify these processes through systemic effects. In obesity and T2DM, AGEs accumulate in the skin, binding to RAGE receptors on keratinocytes and endothelial cells. This activates MAPK and NF-κB pathways, triggering the production of cytokines, chemokines, and adhesion molecules that perpetuate inflammation. AGEs also impair dermal elasticity and microvascular function by cross-linking collagen and elastin, reducing oxygen and nutrient delivery to psoriatic plaques. Hypoxia in plaques stabilizes HIF-1α, which further drives keratinocyte hyperproliferation and AMP expression [49].

Dysregulated lipid metabolism in MetS compounds the problem. Elevated circulating LDL and triglycerides infiltrate the dermis, where they oxidize and activate macrophages via scavenger receptors such as CD36. This enhances the production of TNF-α and IL-1β, creating a pro-inflammatory environment. Simultaneously, reduced ceramides in psoriatic lesions impair epidermal lipid organization, weakening the skin’s ability to prevent microbial colonization and promoting dysbiosis [50].

Therapeutic strategies targeting metabolic dysfunction have shown promise. Weight loss through dietary interventions or bariatric surgery reduces systemic inflammation, normalizes adipokine levels, and improves insulin sensitivity, thereby mitigating psoriatic inflammation. Pharmacologic agents like glucagon-like peptide-1 receptor agonists (GLP-1RAs) might improve psoriasis severity by addressing metabolic abnormalities and meta-inflammation [51]. Experimental interventions targeting lipid metabolism, such as fibrates and PPARγ agonists, demonstrated modest improvements in skin inflammation by restoring microbial equilibrium. These findings suggest that managing lipid dysregulation through microbiota-friendly dietary modifications or lipid-lowering agents could offer a dual benefit in controlling systemic and localized inflammation [47,48].

Microbiome-targeted therapies are emerging as adjunctive treatments for psoriasis. Topical or systemic probiotics that enhance commensal bacteria like *Corynebacterium* and *Cutibacterium* are under investigation. For instance, lactobacilli have shown immunomodulatory effects by promoting regulatory Treg activity and suppressing Th17-driven inflammation. Postbiotics are bioactive compounds produced during the fermentation process by probiotic microorganisms. They include metabolic byproducts such as short-chain fatty acids (SCFAs), bacteriocins, enzymes, vitamins, and other molecules that exert health benefits. Unlike live probiotics, postbiotics are non-living and are considered safer for use in certain populations. They play a role in modulating the microbiome, inhibiting pathogenic microbes, and enhancing host physiological functions, including skin barrier restoration [52].

An emerging area of interest in psoriasis management involves harnessing bacteriophages (phages) to selectively reduce pathogenic bacteria and rebalance the skin microbiome. Recent studies suggest that supplementing lesional skin with phages that target species like *S. aureus* or *Pseudomonas aeruginosa*—microbes frequently implicated in psoriatic dysbiosis—can help decrease inflammation and restore microbial diversity. Practical strategies include topically applied phage cocktails, which may be formulated as creams, ointments, or gels for direct application to psoriatic plaques, as well as oral phage preparations aimed at modulating both the gut and skin microbiota. In preliminary ex vivo and murine models of skin infection, phages effectively reduced bacterial load and lowered levels of pro-inflammatory cytokines, indicating their potential utility in psoriasis. Early anecdotal reports of phage-rich water ingestion (e.g., Ganga river water) have also shown promise in improving psoriasis symptoms for some individuals, although well-designed clinical trials are needed to establish optimal phage combinations, dosing regimens, and delivery methods. If validated, phage therapy could offer a targeted, microbiome-focused adjunct to existing anti-inflammatory and immunomodulatory treatments, potentially improving outcomes in patients who do not respond well to current therapies [53].

These findings highlight the intricate connections between metabolic disorders, the microbiome, and psoriasis, providing new opportunities for integrated treatment approaches that target both systemic and local factors.

## 7. Atopic Dermatitis

AD is a chronic inflammatory skin condition defined by intense itching, eczematous lesions, and impaired epidermal barrier function. Its pathogenesis hinges on mutations in structural proteins like filaggrin and dysregulated Th2 immune responses. Metabolic disorders such as obesity and T2DM exacerbate AD by amplifying systemic inflammation, altering lipid metabolism, and fostering microbial dysbiosis. Together, these factors perpetuate the cycle of barrier dysfunction, immune activation, and microbial imbalance central to AD pathology [54].

The epidermal barrier serves as a critical defense against transepidermal water loss, environmental allergens, and microbial invasion. Filaggrin, a key barrier protein, is crucial for forming the cornified envelope and generating natural moisturizing factors such as urocanic acid and pyrrolidone carboxylic acid, which maintain an acidic skin pH. Mutations in the filaggrin gene, commonly seen in AD, reduce natural moisturizing factor production, impair barrier integrity, and facilitate allergen and microbial penetration, initiating inflammation. Metabolic disorders exacerbate this barrier dysfunction by promoting the accumulation of AGEs. These molecules cross-link keratinocyte and extracellular matrix proteins, stiffening the epidermis and activating RAGE on keratinocytes. RAGE signaling induces NF-κB activation, increasing the production of pro-inflammatory cytokines such as IL-1β, TNF-α, and IL-6, which further impair keratinocyte differentiation and reduce the expression of filaggrin, involucrin, and loricrin [35,36,54].

Th2-driven inflammation dominates AD’s immune landscape, with cytokines like IL-4, IL-5, and IL-13 impairing keratinocyte differentiation and suppressing AMP production. IL-4 and IL-13 further reduce filaggrin expression and promote IgE-mediated allergic sensitization. Chronic inflammation can shift to mixed Th2/Th22 or Th1/Th17 responses, especially in severe or chronic lesions. Metabolic inflammation amplifies these immune dysregulations, with adipokines like leptin promoting Th2 polarization and IL-4/IL-13 production. Simultaneously, adiponectin, an anti-inflammatory adipokine, is reduced in obesity and T2DM, removing a regulatory brake on Th2 activity. This dual effect intensifies AD’s chronic inflammatory state [55].

AD is strongly associated with microbial dysbiosis, characterized by overgrowth of *S. aureus* and diminished diversity of commensal microbes. *S. aureus* plays a direct role in inflammation by producing virulence factors, including superantigens like staphylococcal enterotoxins that activate T cells and amplify Th2 and Th17 responses. Additionally, *S. aureus* secretes proteases, such as V8 protease, which degrade filaggrin and further weaken the epidermal barrier. Its ability to form biofilms enhances resistance to antimicrobial defenses and contributes to persistent inflammation. Hyperglycemia and altered lipid metabolism in metabolic disorders exacerbate *S. aureus* colonization by providing an environment rich in nutrients and substrates, including glucose and free fatty acids. Dysregulated AMP production in AD further weakens microbial control, allowing *S. aureus* to dominate and outcompete commensals [56].

Lipid composition in the stratum corneum is critical for maintaining barrier integrity and microbial balance. Ceramides, cholesterol, and free fatty acids are organized into lamellar structures that prevent TEWL and microbial penetration. Systemic lipid abnormalities in AD, such as increased LDL cholesterol and altered HDL composition, correlate with disease severity and systemic inflammation, as measured by SCORAD scores (r = 0.64, *p* < 0.001). Notably, ceramide abnormalities in the stratum corneum impair the lipid barrier, predisposing the skin to colonization by Staphylococcus aureus and contributing to chronic inflammation. Furthermore, metabolic disorders exacerbate cutaneous lipid abnormalities by influencing sebaceous gland activity and epidermal lipid synthesis. For instance, insulin resistance increases the ratio of saturated to unsaturated fatty acids, favoring colonization by pro-inflammatory microbes like *S. aureus* over commensal species such as *C. acnes*. *S. aureus* lipases further degrade lipids into irritant free fatty acids, aggravating keratinocyte dysfunction and inflammation [57].

Clinically, patients with AD and coexisting metabolic disorders often present with more severe and widespread lesions. Systemic inflammation, oxidative stress, and dysbiosis associated with metabolic dysfunction worsen disease activity and reduce responsiveness to standard treatments like topical corticosteroids and calcineurin inhibitors. However, improved metabolic control can significantly mitigate these effects. Weight loss and glycemic management reduce systemic inflammation, restore AMP production, and improve barrier integrity, thereby decreasing *S. aureus* colonization and inflammation [14].

Emerging microbiome-targeted therapies are gaining attention in AD management. Probiotics, including lactobacilli and *Bifidobacterium* species, enhance microbial diversity and modulate immune responses by increasing Treg activity and reducing Th2 polarization. Postbiotics, such as SCFAs, strengthen the epidermal barrier and suppress *S. aureus* virulence [52].

Recent research demonstrates that bacteriophages can specifically target Staphylococcus aureus—a bacterial species closely linked to AD flares—while preserving beneficial skin microbes such as *S. epidermidis*. In a 2020 study investigating a phage called SaGU1, scientists observed a sharp reduction in S. aureus populations both in vitro and on the skin of atopic mouse models. Notably, SaGU1 did not harm *S. epidermidis*, suggesting a targeted antimicrobial effect. In vitro, *S. aureus* began re-emerging after 14 h, which may reflect the development of phage resistance; however, adding *S. epidermidis* alongside SaGU1 prevented this bacterial rebound, highlighting a potential synergy between beneficial skin flora and phage therapy. Interestingly, in vivo experiments demonstrated that phage therapy alone was effective enough to suppress *S. aureus* overgrowth, with no statistically significant additional benefit observed when *S. epidermidis* was combined with SaGU1. These findings underscore the promise of phage-based treatments in AD, particularly those designed to selectively eliminate pathogenic staphylococci while leaving commensals intact. Future research will clarify the best formulations, dosing strategies, and adjunctive options—such as pairing phages with topical probiotics or supportive skincare regimens—to enhance barrier function and achieve longer-lasting disease control in AD [53].

When combined with metabolic interventions, these approaches offer a holistic strategy for managing AD, addressing both its systemic and localized drivers. This dual focus could lead to more effective, long-term control of AD in patients with metabolic dysfunctions [58].

## 8. Acne

Acne vulgaris is a multifactorial inflammatory condition that primarily affects the pilosebaceous unit. It is clinically characterized by comedones, inflammatory papules, pustules, and nodules. Its pathogenesis is driven by hyperseborrhea, follicular hyperkeratinization, microbial dysbiosis—particularly involving *C. acnes*—and inflammation. Metabolic disorders, such as obesity and insulin resistance, exacerbate acne through systemic and localized mechanisms, including altered sebaceous gland activity, lipid composition changes, immune dysregulation, and microbial shifts, fostering an environment conducive to acne development [59].

Emerging evidence highlights a significant relationship between insulin resistance and acne vulgaris. In a study by Gruszczyńska et al., involving 41 acne vulgaris patients and 47 healthy BMI-matched controls, insulin resistance was assessed using the homeostasis model assessment of insulin resistance (HOMA-IR). The mean HOMA-IR value was significantly higher in the acne group (3.40 ± 1.49) compared to controls (2.34 ± 0.91, *p* < 0.001). Furthermore, 78% of acne patients met the criteria for insulin resistance (HOMA-IR > 2.1), versus 55% in the control group (*p* = 0.026). When the cut-off value was adjusted to 2.69 (as per the Polish population standard), 63% of acne patients and 30% of controls were diagnosed with insulin resistance, further emphasizing the association (*p* = 0.002). Elevated fasting glucose levels were also observed in the acne group (94.88 ± 7.73 mg/dL) compared to controls (79.51 ± 7.18 mg/dL, *p* < 0.001). These findings suggest that insulin resistance may be an independent factor in acne pathogenesis, warranting consideration during diagnosis and treatment [60].

Elevated insulin levels increase the secretion of insulin-like growth factor-1 (IGF-1), which directly enhances sebaceous gland activity. IGF-1 activates the phosphoinositide 3-kinase (PI3K)/Akt and mammalian target of rapamycin complex 1 (mTORC1) pathways, driving sebocyte proliferation and increasing sebum lipid synthesis. The overproduction of sebum provides a nutrient-rich substrate for *C. acnes*, promoting its colonization and metabolic activity. Moreover, IGF-1 suppresses the nuclear receptor peroxisome proliferator-activated receptor gamma (PPAR-γ), disrupting sebocyte differentiation and lipid homeostasis, which further contributes to sebaceous gland dysfunction [60,61].

Additionally, obesity and insulin resistance compound acne-related inflammation through the secretion of pro-inflammatory adipokines like leptin, which promotes Th1 and Th17 polarization and increases cytokines such as interferon-gamma (IFN-γ), IL-17, and IL-22. These cytokines drive keratinocyte hyperproliferation and sebaceous gland dysfunction. Simultaneously, reduced adiponectin removes a critical anti-inflammatory mediator, exacerbating immune activation. Hyperglycemia further promotes the accumulation of AGEs, which bind to RAGE on keratinocytes and immune cells, activating NF-κB and MAPK pathways and amplifying the inflammatory milieu [60,61].

The composition of sebaceous lipids undergoes significant changes in metabolic disorders. Insulin resistance shifts the balance toward a higher ratio of saturated to unsaturated fatty acids, which have pro-inflammatory properties. Saturated fatty acids stimulate keratinocytes via TLR2, promoting cytokine production, including IL-1β and IL-8. These cytokines recruit neutrophils and drive inflammation. Concurrently, oxidative stress associated with hyperglycemia generates lipid peroxidation products, such as malondialdehyde, which disrupt sebaceous lipid profiles, exacerbating inflammation [60,61].

Polycystic ovary syndrome (PCOS), a common metabolic–endocrine disorder, is intricately linked to acne through hyperandrogenism and insulin resistance. Elevated androgens, such as testosterone and dihydrotestosterone, stimulate sebaceous gland hypertrophy and sebum overproduction. Hyperinsulinemia in PCOS amplifies these effects by suppressing sex hormone-binding globulin, increasing the bioavailability of androgens. Sebum in PCOS-associated acne is enriched in squalene and saturated fatty acids, which serve as substrates for *C. acnes* metabolism. These substrates promote the production of pro-inflammatory metabolites, including porphyrins and short-chain fatty acids, which activate the NLRP3 inflammasome in macrophages and keratinocytes. This inflammasome activation triggers the release of IL-1β and IL-18, contributing to the development of inflammatory lesions [62].

Microbial dysbiosis, particularly involving *C. acnes*, plays a pivotal role in acne pathogenesis. As an anaerobic commensal, *C. acnes* thrives in the lipid-rich environment of sebaceous follicles. It metabolizes triglycerides into free fatty acids through lipase activity, disrupting keratinocyte differentiation and increasing follicular permeability. Certain strains of *C. acnes* are highly pro-inflammatory, producing porphyrins that generate ROS and activate the NLRP3 inflammasome, leading to neutrophilic infiltration and the formation of pustules and nodules. Metabolic disorders worsen this dysbiosis by altering sebaceous lipid composition and increasing follicular pH, favoring the dominance of pathogenic *C. acnes* strains. These strains form biofilms, which protect them from AMPs like cathelicidins and defensins, enhancing their resistance to treatments and sustaining chronic inflammation [39].

Sebaceous lipids are not only a nutrient source for *C. acnes* but also modulate the immune response. Free fatty acids such as palmitic and lauric acids activate TLR2 and TLR4 on keratinocytes and immune cells, inducing cytokine production, including IL-8, TNF-α, and IL-6. These cytokines recruit neutrophils and monocytes to the follicle, amplifying inflammation. Lipid peroxidation products like malondialdehyde generated under oxidative stress further enhance inflammatory cascades. Additionally, *C. acnes* utilizes *quorum sensing* to regulate biofilm formation and virulence factor expression, such as lipases, proteases, and hyaluronidases. These enzymes degrade the extracellular matrix and follicular walls, allowing bacterial invasion and perpetuating tissue inflammation [57,63].

Clinically, patients with acne and concurrent metabolic disorders present with more severe and persistent lesions, including nodulocystic acne. These patients are often less responsive to standard therapies, such as retinoids and antibiotics, due to underlying metabolic and microbial dysregulation. Addressing the metabolic contributions to acne is therefore critical. Metformin, a cornerstone treatment for insulin resistance, has shown promise in acne management by reducing mTORC1 signaling, mitigating oxidative stress, and normalizing sebaceous gland activity. Lifestyle interventions like weight loss have been associated with reductions in systemic inflammation and acne severity [64,65,66].

Microbiome-based therapies are emerging as adjunctive treatments for acne. Probiotics are particularly valuable in modulating the immune system and restoring microbial equilibrium. Species such as *Lacticaseibacillus rhamnosus*, *Lactiplantibacillus plantarum*, and *Bifidobacterium breve* have shown potential in enhancing regulatory T-cell (Treg) activity, which is essential for maintaining immune tolerance and reducing the hyper-inflammatory responses typical of acne. These probiotics work by suppressing pro-inflammatory cytokines such as IL-17 and IL-22, which are associated with Th17-driven immune responses, while simultaneously promoting anti-inflammatory cytokines like IL-10. This dual action helps to mitigate the inflammation and hyperkeratinization observed in acne [52,67].

Postbiotics, including short-chain fatty acids (SCFAs) like acetate, propionate, and butyrate, produced by probiotic fermentation, have been shown to exert anti-inflammatory and antimicrobial effects. For instance, SCFAs can inhibit *C. acnes* virulence factors by disrupting its biofilm formation—a critical mechanism that protects the bacteria from host defenses and treatments. Biofilms are known to foster a chronic inflammatory environment by promoting the persistence of pathogenic *C. acnes* strains, making their disruption a key target for acne therapy. In addition, SCFAs modulate keratinocyte differentiation and lipid metabolism, contributing to a healthier skin barrier and reducing the sebum production that fuels *C. acnes* growth [52,67].

Bacteriocins, another class of postbiotics, are antimicrobial peptides produced by probiotics that specifically target pathogenic bacteria without affecting commensal populations. For instance, bacteriocins like nisin and pediocin have demonstrated the ability to selectively inhibit *C. acnes* proliferation. They achieve this by permeabilizing bacterial membranes or interfering with cell wall synthesis, offering a highly targeted approach to microbial regulation [67].

Furthermore, emerging research suggests that probiotics and postbiotics can alter the skin’s pH and lipid composition, creating an environment less conducive to pathogenic bacterial colonization. By promoting the growth of beneficial commensals such as Staphylococcus epidermidis, these interventions not only counteract the dominance of acne-associated *C. acnes* strains but also restore microbial diversity—a hallmark of healthy skin. Preclinical studies have shown that S. epidermidis can antagonize *C. acnes* through the production of antimicrobial peptides, further emphasizing the synergistic role of commensal bacteria in acne management [67].

In addition to their direct effects on the microbiome, probiotics and postbiotics have systemic benefits. Oral administration of probiotics has been linked to reduced systemic inflammation and oxidative stress, factors that exacerbate acne severity. These benefits extend to the regulation of metabolic pathways, including insulin sensitivity and lipid metabolism, which are often dysregulated in individuals with acne [67].

Bacteriophage-based treatments for acne aim to selectively eliminate *C. acnes* without disturbing the broader skin microbiome. Recent murine experiments have shown that injecting bacteriophages targeting *C. acnes* can reduce inflammatory nodules and epidermal thickening in mice, supporting the therapeutic potential of phages as an alternative or complement to antibiotics—especially given growing antibiotic resistance. Topical formulations containing *C. acnes*-lytic phages have also been investigated: semi-solid creams and hydrogels maintained significant phage viability for weeks to months under proper storage conditions (e.g., refrigeration and limited light exposure). Preliminary human trials with topical phage cocktails have demonstrated a dose-dependent reduction in *C. acnes* colonization, suggesting that optimized phage concentrations could be key to improving clinical outcomes. Future research will need to explore long-term efficacy, ideal phage combinations, and potential synergy with established acne therapies such as topical retinoids or benzoyl peroxide. If validated, phage therapy could offer patients a novel, targeted approach to controlling *C. acnes* overgrowth and associated inflammation while preserving beneficial cutaneous microbes [53].

These approaches, combined with metabolic interventions, offer a comprehensive strategy for managing acne, addressing both the systemic and localized drivers of this multifactorial disease [67].

## 9. Rosacea

Rosacea is a chronic inflammatory skin disorder affecting approximately 5% of the global population. It presents as facial erythema, telangiectasia, and inflammatory lesions such as papules and pustules, with severe cases leading to phymatous changes characterized by thickened and irregular skin. Beyond its visible manifestations, rosacea involves complex interactions between immune dysregulation, neurovascular mechanisms, and environmental triggers. Increasingly, the role of the skin microbiome and its interaction with metabolic dysfunctions, such as obesity and dyslipidemia, has come under scrutiny, highlighting their collective contribution to rosacea’s pathogenesis [68].

Rosacea is strongly associated with alterations in the skin microbiome, particularly an overgrowth of *Demodex* mites, including *Demodex folliculorum* and *Demodex brevis*. These mites are found in significantly higher densities on the skin of rosacea patients—ranging from 0.7/cm² in healthy controls to 10.8/cm² in affected individuals. Their exoskeleton and endosymbiotic bacterium *Bacillus oleronius* contribute to inflammation by releasing proteins and heat-shock proteins that activate Toll-like receptor 2 (TLR2) on keratinocytes, triggering innate immune responses. This activation results in the production of cathelicidins, AMPs that are excessively expressed in rosacea and contribute to both the condition’s pro-inflammatory and vasoactive properties. These inflammatory responses are further amplified by metabolic dysfunction. For instance, elevated levels of TNF-α and IL-6 in obesity and MetS enhance microbial overgrowth and contribute to persistent inflammation, creating a feedback loop between dysbiosis and immune activation. Additionally, Staphylococcus epidermidis overgrowth and reduced *C. acnes* abundance are noted, with other species like *Geobacillus* and *Gordonia* correlating with disease severity. These microbial disruptions, alongside environmental and neurological factors, exacerbate skin barrier dysfunction, promote inflammation, and intensify rosacea symptoms, including erythema and telangiectasia [69,70,71].

The neurovascular component of rosacea intricately interfaces with the microbiome through activation of transient receptor potential (TRP) channels, particularly TRPV1 and TRPA1. These channels, expressed on sensory neurons, keratinocytes, and immune cells, are highly sensitive to microbial metabolites such as SCFAs, lipopolysaccharides, and other bacterial byproducts. Environmental stimuli, including ultraviolet (UV) radiation, heat, and dietary triggers (e.g., capsaicin and alcohol), further potentiate their activation. Upon activation, TRPV1 and TRPA1 stimulate the release of pro-inflammatory neuropeptides, such as substance P and calcitonin gene-related peptide (CGRP), which contribute to neurogenic inflammation. This cascade disrupts neurocutaneous signaling, impairing the production of AMPs such as cathelicidins and β-defensins. AMPs are critical for maintaining microbial homeostasis by directly inhibiting pathogenic bacterial growth and preserving skin barrier integrity. Dysregulated signaling creates a permissive environment for microbial overgrowth, particularly of *Demodex folliculorum*, which harbors symbiotic bacteria such as *Bacillus oleronius*. These bacteria release antigens that further amplify inflammation by activating Toll-like receptor 2 (TLR2) on keratinocytes and immune cells. Additionally, the altered microenvironment fosters the growth of opportunistic pathogens, including *S. epidermidis* and *C. acnes*. The interaction between these microbial populations and the impaired neurovascular signaling exacerbates inflammatory pathways, disrupts skin barrier function, and reinforces the chronicity of rosacea. Importantly, the feedback loop of inflammation, microbial imbalance, and neurovascular dysfunction underscores the need for targeted therapies addressing both microbial and neurovascular components in rosacea management [69,70].

Cutaneous barrier dysfunction in rosacea exacerbates microbial dysbiosis. Downregulation of the ABCA12 gene, a key regulator of lipid lamellae formation, has been observed in rosacea patients. This defect compromises the skin barrier, increasing transepidermal water loss and facilitating microbial infiltration. Dyslipidemia further alters the skin’s lipid environment. Elevated LDL and triglycerides, along with reduced HDL, shift the sebaceous lipid composition, favoring conditions conducive to pathogenic growth. For example, *S. epidermidis*, typically a commensal, may adopt pathogenic traits in response to an altered lipid environment, while oxidative modifications of lipids, such as oxidized LDL (ox-LDL), activate nucleotide-binding oligomerization domain-like receptor protein 3 (NLRP3) inflammasome pathways, sustaining inflammation [57,72].

Obesity and dyslipidemia intensify these microbiome alterations. Adipokines, such as leptin and resistin, secreted by hypertrophic adipose tissue in obesity, enhance Th1 and Th17 polarization, contributing to pro-inflammatory cytokine production that disrupts microbial homeostasis. Reduced adiponectin, a key anti-inflammatory adipokine, further exacerbates this imbalance [23]. Hyperglycemia, commonly associated with MetS, increases nutrient availability for microbial growth, promoting overgrowth of inflammatory taxa. AGEs formed under hyperglycemic conditions interact with RAGE receptors on keratinocytes and immune cells, amplifying NF-κB-driven inflammation and contributing to microbial dysbiosis [73].

A case–control study investigated the connection between rosacea, insulin resistance, and MetS in 47 rosacea patients and 50 controls. Results indicated that IR was significantly higher among rosacea patients (44.7%) compared to controls (20%) (*p* = 0.009), while the prevalence of MS did not differ significantly between the groups (*p* = 0.186). Rosacea patients exhibited elevated levels of fasting blood glucose, LDL, triglycerides, total cholesterol, CRP, and both systolic and diastolic blood pressure compared to controls (*p* < 0.05). These findings underscore the potential role of systemic metabolic dysfunctions, including insulin resistance, in the pathogenesis of rosacea and its associated cardiovascular risks. Notably, markers of inflammation, such as elevated cathelicidin LL-37 levels, oxidative stress, and endoplasmic reticulum stress, are implicated in both rosacea and metabolic disorders, suggesting overlapping pathogenic pathways. These results highlight the importance of evaluating IR in rosacea patients, particularly for those at risk of metabolic and cardiovascular comorbidities [73].

Regarding microbiome-targeted therapies, as already stated, rosacea is linked to the overexpression of TLR2 receptors, which drive inflammatory responses and disrupt the skin microbiome. While scalp rosacea has been managed successfully with a combination of oral probiotics and doxycycline, the potential of topical probiotics for treating rosacea remains unexplored and warrants further investigation [71].

Additionally, systemic metabolic control is critical for addressing the microbiome–metabolism–skin axis in rosacea. Weight loss and dietary interventions targeting dyslipidemia and hyperglycemia have demonstrated reductions in systemic inflammation, normalization of lipid profiles, and improved skin outcomes. Pharmacologic agents like metformin and statins not only improve metabolic parameters but also indirectly enhance microbial diversity by restoring AMP production and reducing inflammatory drivers of dysbiosis. These integrated approaches, combining microbiome-targeted therapies and metabolic interventions, represent a holistic strategy for managing rosacea and mitigating its systemic comorbidities [72].

## 10. Seborrheic Dermatitis

SD is a chronic inflammatory skin disorder that affects 1–3% of the population. It is characterized by erythematous, scaly lesions predominantly localized to sebaceous gland-rich areas such as the face, scalp, and chest. SD’s pathogenesis involves a multifaceted interaction of sebaceous gland activity, microbial dysbiosis—particularly involving *Malassezia* species—and immune system dysregulation. These localized factors are significantly influenced by systemic metabolic conditions such as dyslipidemia and MetS, highlighting the interplay between systemic metabolic health and skin microbiota in SD development [41].

Sebaceous gland activity plays a central role in SD by producing sebum enriched in triglycerides, free fatty acids, wax esters, cholesterol, and squalene, which provide substrates for *Malassezia*. Through lipase activity, *Malassezia* species metabolize these lipids into pro-inflammatory free fatty acids that irritate keratinocytes, inducing the release of IL-6 and IL-1β. These cytokines drive inflammation, disrupt lipid organization in the stratum corneum, and weaken the epidermal barrier, perpetuating clinical symptoms such as scaling, redness, and skin peeling. The inflammatory microenvironment also stimulates abnormal keratinocyte proliferation, further disrupting the epidermal barrier. Although increased sebum production was historically considered essential in SD, newer evidence suggests that seborrhea is not strictly required, underscoring the importance of microbiota and immune factors in disease pathogenesis [51,57].

Altered lipid metabolism contributes significantly to SD’s pathology. Skin lipidomics studies have revealed unique profiles in SD lesions compared to healthy skin, with older research identifying decreased squalene, wax esters, and free fatty acids alongside increased cholesterol and triglycerides in lesional skin. These findings suggest disrupted lipid homeostasis due to abnormal keratinization and sebaceous gland dysfunction. Recent work has shown higher mean skin surface lipid levels in SD patients, correlating with disease severity. Systemic lipid abnormalities, including elevated total cholesterol, LDL, and reduced HDL, are also common in SD patients, with studies linking these dyslipidemias to increased SD severity. Reduced HDL, in particular, diminishes its antimicrobial functions, allowing *Malassezia* overgrowth, sustaining inflammation, and exacerbating dysbiosis [57].

*Malassezia* species are central to SD, with their lipase-driven metabolism of sebaceous lipids fueling inflammation. By hydrolyzing triglycerides into free fatty acids, *Malassezia* disrupts keratinocyte membranes and activates PRRs, such as TLR2, triggering cytokine production and neutrophil recruitment. Different species, including *M. globosa* and *M. restricta*, exhibit variations in lipase activity and immune interactions, leading to site-specific manifestations. In SD-affected skin, microbial dysbiosis is evident, with reduced bacterial diversity and a dominance of *Malassezia*. This imbalance disrupts the competitive effects of commensal bacteria, such as *S. epidermidis*, further enabling *Malassezia* colonization. Additionally, altered ceramide and squalene levels in SD create a lipid environment favorable for *Malassezia* proliferation, exacerbating inflammation [74].

The exaggerated inflammatory response in SD involves the upregulation of cytokines like TNF-α, IL-6, and IL-1β, alongside activation of NF-κB and MAPK pathways. These inflammatory signals promote keratinocyte hyperproliferation, barrier dysfunction, and scaling. MetS and dyslipidemia amplify these immune dysregulations, with hypertriglyceridemia and elevated LDL levels activating TLRs on keratinocytes and macrophages. This activation promotes further cytokine release and oxidative stress, creating a feedback loop between systemic lipid imbalances and local skin inflammation. Reduced HDL removes a critical anti-inflammatory factor, worsening the inflammatory cascade and microbial dysbiosis in SD [75].

Treatment of SD traditionally focuses on managing inflammation and *Malassezia* overgrowth. Topical antifungal agents, such as ketoconazole and ciclopirox, and anti-inflammatory therapies, such as calcineurin inhibitors and corticosteroids, form the cornerstone of therapy. For severe cases, oral antifungals like itraconazole or systemic retinoids may be required. However, systemic metabolic factors offer additional therapeutic targets. Weight loss and dietary interventions improve lipid profiles and reduce systemic inflammation, indirectly benefiting SD. Statins, which lower LDL and exhibit anti-inflammatory effects, could serve as an adjunct therapy in SD patients with significant dyslipidemia or MetS [76].

Emerging therapies targeting lipid metabolism and the skin microbiome present exciting opportunities for SD management. Topical formulations restoring ceramides and squalene levels may strengthen the epidermal barrier and reduce *Malassezia* colonization. Probiotics and postbiotics, aimed at enhancing microbial diversity, can suppress inflammation and dysbiosis. For instance, probiotics such as lactobacilli and *Bifidobacterium* species modulate immune responses and increase commensal bacterial populations, countering *Malassezia* overgrowth. Postbiotic compounds, including SCFAs, may further stabilize microbial communities and reduce cytokine-driven inflammation [77,78].

Specifically, a recently published study evaluated the efficacy of EUTOPLAC, a probiotic-enriched oily suspension containing Lactobacillus crispatus P17631 and Lacticaseibacillus paracasei I1688, in modulating the skin mycobiome–bacteriome and alleviating SD symptoms. The study enrolled 25 patients with moderate to severe SD who applied EUTOPLAC daily for one week. Symptom severity, assessed using the Seborrheic Dermatitis Area Severity Index (SDASI), significantly improved, with mean SDASI scores decreasing from baseline (T0) to T8 (*p* < 0.0001). Improvements persisted three weeks post-treatment (T28), though scores plateaued between T8 and T28. Fungal diversity, measured via Shannon and Pielou indices, significantly increased at T8 (Shannon index: 2.66 ± 0.99 vs. T0: 1.37 ± 0.07; *p* < 0.0001), accompanied by a reduction in Malassezia relative abundance (74.8% at T8 vs. 96.5% at T0; *p* < 0.0001). By T28, Malassezia abundance partially rebounded to 93.0%. Bacteriome analysis showed a marked rise in Lactobacillus and Lacticaseibacillus genera at T8 (23.1% and 0.6%, respectively, vs. 0.1% and 0.003% at T0; *p* < 0.0001), accompanied by a significant decrease in Staphylococcus (6.9% at T8 vs. 28.7% at T0; *p* < 0.0001). These effects were transient, normalizing by T28. Network analysis revealed significant disruptions in microbial correlations at T8, reflecting transient community instability post-treatment. These findings highlight EUTOPLAC’s potential as a targeted SD therapy, offering temporary symptom relief and microbiome modulation without long-term dysbiosis. Further randomized controlled trials are needed to confirm these benefits and optimize probiotic formulations [77].

These integrative approaches, targeting both systemic and local factors, hold promise for improving outcomes in SD while addressing its complex pathophysiology [76,77,78].

## 11. Hidradenitis Suppurativa

HS is a chronic inflammatory skin condition primarily affecting intertriginous areas, including the armpits, groin, submammary folds, and perianal regions. It is characterized by recurrent painful nodules, abscesses, fistulae, and significant scarring, with a profound impact on quality of life. While HS is often underdiagnosed, recent advances have illuminated its complex pathogenesis, linking follicular occlusion, dysregulated immunity, microbial dysbiosis, and systemic metabolic disturbances. A notable connection between HS and MetS has underscored the role of the microbiome as a mediator between metabolic dysfunction and the chronic inflammation observed in HS [15,79].

The initial event in HS involves follicular hyperkeratosis, resulting in the plugging and dilation of pilosebaceous units. Follicular rupture releases keratin and sebum into the dermis, triggering a robust inflammatory response. Both innate and adaptive immunity play critical roles, with elevated levels of IL-1β, IL-17, TNF-α, and IL-12/23 perpetuating inflammation. Dysbiosis of the skin microbiome significantly amplifies these responses, with lesional areas dominated by pathogenic bacteria such as *S. aureus*, *S. pyogenes*, and *C. acnes*. Biofilms formed by these pathogens further protect them from immune clearance and antimicrobial treatments, fostering chronicity [15,79].

The correlation between MetS and HS is well documented, with an estimated odds ratio of HS patients for MetS development being 2.66 (95 CI: 1.90–3.72) [15]. Metabolic dysfunction exacerbates this dysbiosis, creating a skin environment conducive to pathogenic microbial overgrowth. Elevated glucose levels associated with hyperglycemia provide abundant nutrients for bacterial proliferation, while dysregulated sebaceous lipid profiles favor the colonization of pathogenic strains over commensals. Hyperinsulinemia and insulin resistance, both hallmarks of MetS, activate the mammalian target of rapamycin complex 1 signaling pathway, promoting keratinocyte hyperproliferation and follicular plugging, thus aggravating HS lesions. Inflammatory cytokines such as TNF-α and IL-17, elevated in both HS and metabolis syndrome, disrupt AMP production, further compromising microbial homeostasis and exacerbating dysbiosis [15,80].

Obesity, particularly abdominal obesity, is strongly associated with HS severity, as visceral adipose tissue secretes pro-inflammatory adipokines like leptin and resistin, which promote Th1 and Th17 polarization. Reduced levels of adiponectin, an anti-inflammatory adipokine, remove a critical regulatory mechanism against inflammation, intensifying systemic and localized immune dysregulation. Hypertriglyceridemia and reduced HDL in HS patients reflect a pro-atherogenic lipid profile, while inconsistent findings on LDL and total cholesterol levels highlight the need for further research. The involvement of metabolic dysfunction-associated steatotic liver disease in HS patients further underscores the systemic metabolic impact of the disease [15,80].

Skin lipidomics studies have provided key insights into the metabolic interplay in HS. Lesional skin in HS exhibits an imbalance between pro- and anti-inflammatory lipid mediators, with elevated omega-6 fatty acid metabolites derived from 5-lipoxygenase (5-LO) and reduced levels of omega-3 fatty acids such as docosahexaenoic acid (DHA). This shift in lipid mediator profiles perpetuates localized inflammation and delays resolution. Additionally, elevated triglycerides and reduced HDL-C levels commonly observed in HS patients disrupt lipid organization in the skin’s sebaceous environment, facilitating pathogenic colonization and biofilm formation [57].

Microbial dysbiosis in HS is marked by reduced diversity and overrepresentation of pathogenic taxa. These microbial shifts are closely tied to the inflammatory microenvironment in HS lesions. *C. acnes*, a facultative anaerobe, metabolizes sebaceous triglycerides into free fatty acids, which disrupt keratinocyte membranes and trigger inflammatory responses via TLR2. Biofilms produced by *C. acnes* and other pathogenic bacteria shield these microbes from host immune defenses and antimicrobial treatments, enabling sustained inflammation and tissue damage. Dysbiosis-associated microbial metabolites, including SCFAs and ROS, exacerbate keratinocyte dysfunction and further perpetuate the inflammatory cycle [81].

Therapeutic strategies for HS must address the interactions between the skin microbiome, metabolic dysfunction, and chronic inflammation. Standard treatments, including TNF-α inhibitors like adalimumab and IL-17 or IL-12/23 inhibitors such as secukinumab and ustekinumab, target key inflammatory pathways but do not address underlying metabolic contributors. Weight loss through lifestyle interventions or bariatric surgery has been shown to improve HS severity, likely by reducing systemic inflammation and restoring metabolic balance [15,80].

According to this systematic review, GLP1-RAs, particularly liraglutide and semaglutide, demonstrate significant therapeutic potential in managing HS. Liraglutide, administered at 3 mg for three months in a prospective case series, resulted in a reduction in BMI from 39.3 ± 6.2 to 35.6 ± 5.8 (*p* = 0.002), waist circumference from 121.3 ± 19.2 cm to 110.6 ± 18.1 cm (*p* = 0.01), and C-reactive protein (CRP) levels from 4.5 ± 2.2 mg/L to 3.0 ± 2.1 mg/L (*p* = 0.04). Significant improvements in HS severity were observed, with Hurley stage scores decreasing from 2.6 ± 0.5 to 1.1 ± 0.3 (*p* = 0.002), and Dermatology Life Quality Index (DLQI) scores improving from 12.3 ± 2.8 to 9.7 ± 6.9 (*p* = 0.04). Semaglutide was similarly associated with weight reduction (mean body weight decrease from 117.7 kg to 111.6 kg; *p* < 0.001) and improved quality of life (DLQI score reduction from 13 to 9; *p* = 0.001). However, reductions in HS flare frequency and CRP levels did not reach statistical significance. These findings support the role of GLP1-RAs in addressing both metabolic and inflammatory components of HS, warranting further investigation in controlled trials [82].

Microbiome-targeted therapies are emerging as adjuncts in HS management. Probiotics, such as lactobacilli and *Bifidobacterium* strains, enhance microbial diversity and modulate immune responses by increasing Treg activity and suppressing Th17-driven inflammation. Postbiotics, including SCFAs, directly inhibit pathogenic biofilm formation and virulence while promoting commensal populations. These approaches, combined with systemic metabolic interventions, provide a comprehensive strategy for addressing both the localized and systemic drivers of HS, highlighting the central role of the skin microbiome in mediating metabolic interactions in this debilitating condition [81].

Lifestyle interventions, including weight loss, smoking cessation, and dietary adjustments, play a significant role in managing HS. These modifications not only have the potential to alleviate disease symptoms but also influence microbial diversity. For example, smoking has been associated with an increase in the gut phyla Proteobacteria and Bacteroidetes while reducing Actinobacteria and Firmicutes. Smoking also lowers overall gut microbiome diversity through mechanisms such as oxidative stress, alterations in mucin composition, and disruption of intestinal tight junctions. Consequently, smoking cessation may restore microbial balance, providing an additional pathway for therapeutic benefit. Probiotics have emerged as a promising adjunctive treatment in HS due to their ability to restore microbial homeostasis, particularly by increasing beneficial skin bacteria such as *Cutibacterium* spp., *Corynebacterium*, and *Staphylococcus*. Targeting prelesional skin with topical probiotics or using oral formulations to modulate gut microbiota offers potential therapeutic advantages. Probiotics can impact systemic inflammation, oxidative stress, glycemic control, and lipid metabolism, all of which are implicated in HS pathogenesis. While extensive evidence supports the use of probiotics in conditions like AD and psoriasis, their role in HS is still underexplored. More studies are needed to identify the most effective strains and their mechanisms. Dietary interventions, such as reducing high-fat and high-sugar intake, avoiding dairy, and eliminating brewer’s yeast, may also benefit HS patients. However, the full impact of diet on HS severity remains insufficiently understood, necessitating further research to establish evidence-based dietary recommendations. Together, these lifestyle and dietary approaches could pave the way for personalized HS management strategies [81].

**Figure 1:**
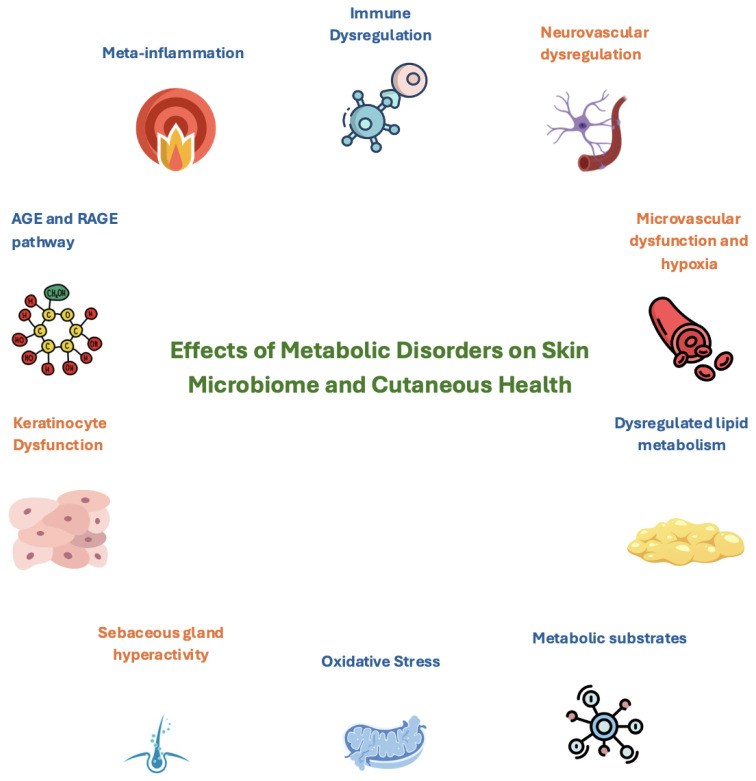
Interconnected pathways linking metabolic disorders to skin microbiome dysbiosis and cutaneous disease. Metabolic disorders influence skin health and the microbiome through interconnected mechanisms. Chronic low-grade inflammation, or meta-inflammation, driven by pro-inflammatory cytokines such as TNF-α, IL-6, and IL-1β, disrupts keratinocyte differentiation, weakens the epidermal barrier, and alters AMP production, increasing susceptibility to infections and dysbiosis. Immune dysregulation plays a significant role, as adipokines like leptin promote Th1/Th17 polarization, while reduced adiponectin removes anti-inflammatory control, intensifying immune activation and microbial imbalances. Neurovascular dysregulation, a notable mechanism in rosacea, is driven by the increased activation of pathways such as TRPV1 channels and exacerbates skin sensitivity and dysbiosis. Microvascular dysfunction and reduced capillary perfusion create hypoxic conditions that favor anaerobic or facultative anaerobic microbes, altering microbial ecology. Dysregulated lipid metabolism, particularly altered sebaceous gland activity in insulin resistance, leads to changes in sebum composition, such as increased saturated fatty acids, which promote the colonization of pathogenic microbes and disrupt the balance of commensal microbes. Systemic nutritional and metabolic influences, including hyperglycemia and dyslipidemia, provide substrates for microbial growth, destabilizing skin homeostasis. Oxidative stress and lipid peroxidation further damage keratinocytes and lipids, compromising skin integrity and promoting microbial overgrowth. Sebaceous gland hyperactivity, induced by hyperinsulinemia and IGF-1, stimulates excessive lipid production, creating a nutrient-rich environment for opportunistic microbes. Cytokines and oxidative stress reduce the expression of barrier proteins like filaggrin and involucrin, increasing transepidermal water loss and weakening physical defenses against microbial invasion. AGEs, formed under hyperglycemic conditions, bind to their receptor RAGE, triggering NF-κB-mediated inflammation and oxidative stress. This process impairs skin barrier proteins, disrupts collagen cross-linking, and affects keratinocyte function. These mechanisms collectively illustrate how metabolic disorders create both systemic and localized environments conducive to skin dysbiosis, inflammation, and disease, underscoring the need for integrated therapeutic strategies targeting metabolic dysfunction and skin health. Systemic effects are marked in blue, while localized effects are marked in orange.

**Figure 2:**
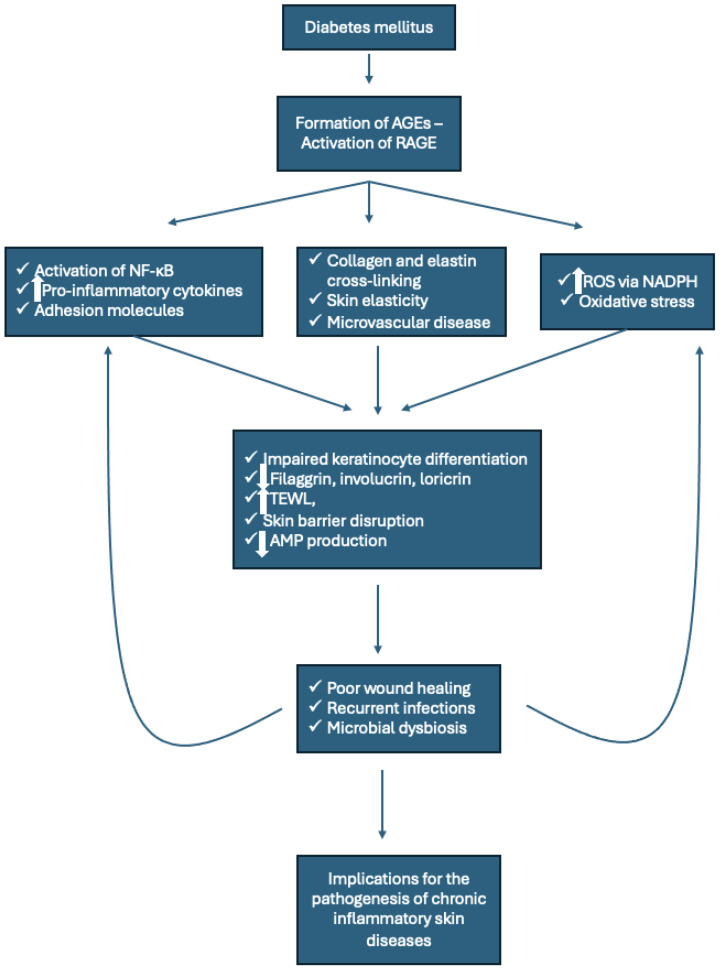
Flowchart of the complex interactions between AGE-RAGE pathway and the skin. Abbreviations. AGEs (Advanced Glycation End Products); AMP (Antimicrobial Peptides); NF-κB (Nuclear Factor Kappa-Light-Chain-Enhancer of Activated B cells); RAGE (Receptor for Advanced Glycation End Products); ROS (Reactive Oxygen Species); TEWL (Transepidermal Water Loss); ↑ (increased); ↓ (decreased).

A schematic illustration of the effects of Metabolic Disorders on Skin Microbiome and Cutaneous Health is presented in Figure 1. An overview of the correlations between skin diseases, microbiota shifts, metabolic influences, and therapeutic opportunities is presented in Table 2. A flowchart of the complex interactions between AGE-RAGE pathway and the skin is illustrated in Figure 2.

## 12. Integrated Therapeutic Implications and the Role of ML

Comprehensive management of metabolic disorders—through lifestyle modifications, pharmacotherapy (e.g., metformin, sodium–glucose cotransporter-2 inhibitors, glucagon-like peptide-1 receptor agonists), and sometimes bariatric surgery—can alleviate systemic inflammation and modulate skin lipid profiles. Improved metabolic control can restore normal AMP production, reduce AGE formation, and promote commensal dominance over pathogenic taxa [5,9]. Additionally, interventions that enhance insulin sensitivity and decrease adipose-derived inflammation can indirectly stabilize the cutaneous immune environment, making it less susceptible to microbial imbalances. These systemic strategies not only reduce skin inflammation but also improve the efficacy of topical treatments. When local therapeutics fail to eradicate problematic microbes completely, the improved systemic environment ensures that the skin becomes less favorable for pathogenic colonization. Over time, this integrated approach may reduce the recurrence of inflammatory skin conditions and infections, resulting in improved long-term patient outcomes.

Therapeutic strategies targeting the skin microbiota, such as topical prebiotics, probiotics, or bacteriophage therapy, represent direct methods to restore microbial equilibrium [77,83,84,85]. Advances in postbiotic formulations (e.g., microbial metabolites like short-chain fatty acids, bacteriocins, and bioactive peptides) have shown promise in enhancing skin barrier function, increasing AMP activity, and reducing pro-inflammatory signaling pathways. For example, SCFAs, derived from probiotic fermentation, not only suppress pathogenic biofilm formation but also enhance keratinocyte differentiation and lipid metabolism. These treatments are particularly beneficial when combined with systemic metabolic interventions, ensuring a synergistic effect that improves skin health while addressing the root causes of dysbiosis [86]. By simultaneously correcting underlying metabolic disturbances and stabilizing microbial communities, clinicians may achieve more durable and long-lasting improvements in the cutaneous ecosystem. This approach is especially critical for conditions prone to relapse, where fostering a stable, health-promoting microbial community reduces the frequency and intensity of disease flares.

Recent advancements in understanding the skin microbiome have unveiled significant insights into its role in immune regulation and host–microbe interactions. A study by Bousbaine et al. (2024) highlights the skin’s ability to generate a potent, durable antibody response against Staphylococcus epidermidis, a common commensal. This immune response, which involves both CD8+ T cells and antibodies, specifically targets the surface protein Aap of S. epidermidis, offering potential for novel topical vaccination strategies. By engineering S. epidermidis strains to express immunogenic proteins, researchers were able to elicit strong antibody responses, including robust IgA in mucosal sites, under physiologic colonization conditions. Meanwhile, research by Gribonika et al. (2024) further expands the role of the skin as an autonomous lymphoid organ, where microbial colonization triggers dual immune responses. Skin commensals promote the formation of classical germinal centers in lymph nodes for systemic IgG responses and induce the development of tertiary lymphoid organs within the skin to locally produce IgG antibodies, effectively controlling microbial biomass and preventing systemic infection. Together, these studies underscore the skin’s dynamic immune functions, revealing its capacity for both local and systemic regulation of microbial symbiosis and potential pathogenesis [87,88].

These findings hold significant implications for the development of therapeutics targeting the skin microbiome. By harnessing the skin’s ability to generate both localized and systemic immune responses, it may be possible to design innovative treatments that leverage the skin’s natural immunological processes. For instance, engineered *S. epidermidis* strains, as described by Bousbaine et al. [87] could be used in topical vaccines that stimulate robust immune responses without the need for systemic interventions. Additionally, the identification of skin-specific immune mechanisms, as shown by Gribonika et al. [88] offers potential for localized therapeutic strategies aimed at modulating microbial interactions and controlling inflammation or infection directly at the skin barrier. These insights provide a promising framework for developing microbiome-based therapies to address a range of dermatological and systemic diseases.

Despite these advances, the clinical translation of combined metabolic and microbiota-targeted therapies faces significant challenges. Variability in patient responses to probiotics or postbiotics, stemming from differences in baseline microbiota composition, genetic predispositions, and environmental exposures, complicates the standardization of these treatments. Regulatory hurdles, including inconsistent definitions and approval pathways for probiotics and postbiotics, further hinder their widespread clinical use. Additionally, the lack of long-term studies evaluating the safety and efficacy of these interventions limits their integration into standard practice. Addressing these barriers requires robust clinical trials that explore personalized approaches, optimize therapeutic formulations, and assess the sustainability of microbiota modulation over time.

Emerging ML technologies provide tools to overcome many of these challenges by integrating complex datasets to predict patient-specific responses and guide treatment decisions. ML algorithms, using multi-omic datasets such as metabolomics, lipidomics, microbiome profiles, and clinical records, can uncover previously unrecognized microbial signatures correlated with metabolic parameters and disease outcomes. For example, supervised learning models (e.g., random forests, gradient boosting machines) can predict which patients will benefit most from probiotic or postbiotic interventions based on their baseline microbiome composition. Deep learning models go further by integrating temporal data, identifying prognostic biomarkers, and differentiating between inflammatory skin conditions with overlapping clinical features. These computational tools enable clinicians to tailor interventions precisely, reducing trial-and-error prescribing and improving outcomes [10,11,89].

Furthermore, ML-driven biomarker discovery can enhance the correlation between molecular biomarkers—such as serum cytokines, microbial taxa, or lipid profiles—and clinical outcomes. Identifying predictive biomarkers not only ensures that metabolic and microbiome-directed therapies are applied to patients most likely to benefit but also facilitates earlier interventions that could prevent disease progression. As these biomarkers are validated, they can be incorporated into clinical decision support systems, allowing physicians to predict treatment responses with greater confidence and refine therapeutic protocols for individual patients. Over time, these advancements may improve the precision and personalization of dermatological care, particularly for individuals with underlying metabolic dysfunctions [89,90,91].

The integration of ML into clinical practice is not without its challenges. Data heterogeneity, the need for standardization across multi-omics platforms, and ethical considerations related to patient data privacy must be addressed to fully realize the potential of these technologies. Nonetheless, as more data become available and ML models grow more accurate, their capacity to unravel the complex interplay between metabolic disorders, the microbiota, and skin health will only expand, enabling transformative advances in personalized medicine.

## 13. Conclusions and Future Directions

In conclusion, this review highlights the intricate interplay between metabolic disorders, skin microbiota, and dermatologic health, emphasizing the systemic drivers of inflammatory skin conditions such as psoriasis, atopic dermatitis, and acne. Metabolic dysregulation profoundly affects microbial equilibrium, immune responses, and skin barrier function, contributing to disease onset and progression.

Comprehensive management strategies that address metabolic dysfunction—through lifestyle modifications, pharmacological interventions like GLP-1 receptor agonists and SGLT-2 inhibitors, and bariatric surgery—can mitigate systemic inflammation and restore microbial homeostasis. Concurrently, microbiome-targeted therapies, including probiotics, prebiotics, postbiotics, and bacteriophage treatments, have shown promise in re-establishing microbial balance and enhancing barrier integrity. Integrating these approaches with ML models allows for the identification of predictive biomarkers and enables the development of highly personalized, effective therapeutic strategies.

Future research must focus on overcoming clinical translation barriers, such as patient variability, regulatory challenges, and the lack of long-term studies on microbiota-targeted therapies. High-resolution, longitudinal studies integrating multi-omics datasets will be essential to elucidate host–microbiome dynamics. Interdisciplinary collaboration across dermatology, endocrinology, microbiology, and computational biology will be crucial in advancing precision medicine. These efforts aim to create durable, patient-specific treatments that bridge systemic and localized therapies, ultimately improving outcomes and reducing the burden of skin diseases influenced by metabolic dysfunction.

## Figures and Tables

**Table 1:** Roles of commensal and pathogenic bacteria in skin barrier modulation.

Barrier Type	Commensal Role	Pathogen Role	References
Physical Barrier	- *S. epidermidis*: produces AMPs, strengthens keratinocyte adhesion. - *Corynebacterium*: supports skin integrity by interacting with lipids.	- *S. pyogenes*: produces proteases, disrupting structural proteins. - *S. aureus*: forms biofilms, aiding invasion.	[4,28,29,30,31,32]
Chemical Barrier	- *Corynebacterium* and *Cutibacterium*: contribute to sebum metabolism, producing fatty acids with antimicrobial properties. - Lactobacilli: enhance AMP production like defensins.	- *S. aureus*: reduces AMP production (e.g., β-defensins). - *S. pyogenes*: secretes exotoxins, amplifying inflammation.	
Immune Barrier	- *Bifidobacterium*: modulates Tregs, promoting immune tolerance. - Lactobacilli: reduce Th2/Th17-driven inflammation, balancing immune responses.	- *S. aureus*: secretes superantigens, driving T-cell activation. - *S. pyogenes*: triggers excessive immune responses.	
Lipid Barrier	- *Cutibacterium:* maintain an acidic environment, modulate lipid metabolism. - Lactobacilli: stabilize lipid profiles, supporting barrier function.	- *S. aureus*: alters lipid profiles, promoting inflammation. - *C. acnes* (pathogenic strains): produces porphyrins, triggering inflammation.	
Microenvironmental Factors	- *Corynebacterium* and *Bifidobacterium*: regulate pH and microbial balance. - Lactobacilli: maintain a low pH, deterring pathogenic growth.	- *S. pyogenes* and *S. aureus*: create oxidative stress, damaging lipids and proteins. - *Malassezia*: disrupts lipid homeostasis.	

**Table 2:** Correlation between skin diseases, microbiota shifts, metabolic influences, and therapeutic opportunities.

Disease	Key Microbiota Shifts	Metabolic Influences	Therapeutic Opportunities	References
Psoriasis	Reduced *Cutibacterium* and *Corynebacterium*, increased *S. aureus* and *S. pyogenes*.	Obesity-induced inflammation (elevated TNF-α, IL-6), dyslipidemia, and advanced glycation end products (AGEs).	Weight loss, GLP-1 receptor agonists, probiotics (e.g., Lactobacillus species), postbiotics (SCFAs), topical and systemic anti-inflammatory agents, microbiome restoration therapies.	[44,45,46,47,48,49,50,51,52,53]
Atopic Dermatitis	Overgrowth of *S. aureus*, reduced microbial diversity.	Hyperglycemia, AGE formation, adipokine dysregulation (e.g., elevated leptin, reduced adiponectin).	Improved metabolic control (e.g., glycemic management), probiotics (e.g., lactobacilli and *Bifidobacterium*), postbiotics, topical prebiotics, barrier repair therapies (e.g., ceramide-based creams).	[14,54,55,56,57,58]
Acne	Dysbiosis with overrepresentation of pathogenic *C. acnes* strains.	Insulin resistance, IGF-1 elevation, sebaceous gland dysregulation (altered lipid profiles).	Metformin, lifestyle interventions (e.g., reduced glycemic load), probiotics (*Lacticaseibacillus rhamnosus*), postbiotics, anti-inflammatory agents targeting sebaceous activity, topical retinoids.	[59,60,61,62,63,64,65,66,67]
Rosacea	Increased *Demodex folliculorum*, reduced microbial diversity; overgrowth of *S. epidermidis*.	Dyslipidemia, oxidative stress, systemic inflammation (e.g., elevated TNF-α, IL-6).	Weight loss, statins for dyslipidemia, probiotics (*Lacticaseibacillus rhamnosus*), postbiotics (e.g., SCFAs), topical agents targeting Demodex and reducing neurogenic inflammation (e.g., calcineurin inhibitors).	[23,57,68,69,70,71,72,73]
Seborrheic Dermatitis	Dominance of *Malassezia* species, reduced bacterial diversity.	Dyslipidemia, altered sebaceous lipid metabolism, systemic inflammation.	Antifungal agents (e.g., ketoconazole), ceramide-based barrier repair, probiotics to restore microbial diversity, dietary interventions to improve lipid profiles, anti-inflammatory therapies (e.g., calcineurin inhibitors).	[41,51,57,74,75,76,77,78]
Hidradenitis Suppurativa	Reduced diversity, overgrowth of *S. aureus*, *S. pyogenes*, and *C. acnes*.	Obesity, hyperinsulinemia, elevated IL-17/TNF-α, dyslipidemia.	GLP-1 receptor agonists (e.g., liraglutide), weight loss, targeted probiotics, postbiotics, biologics (e.g., TNF-α inhibitors), lifestyle interventions to address metabolic dysregulation.	[15,57,79,80,81,82]

Abbreviations. AGEs (Advanced Glycation End Products); GLP-1 (Glucagon-Like Peptide-1); IL (Interleukin); IGF-1 (Insulin-Like Growth Factor-1); SCFAs (Short-Chain Fatty Acids); TNF-α (Tumor Necrosis Factor-Alpha).
